# Identification of phenolic secondary metabolites from* Schotia brachypetala* Sond. (Fabaceae) and demonstration of their antioxidant activities in *Caenorhabditis elegans*

**DOI:** 10.7717/peerj.2404

**Published:** 2016-11-15

**Authors:** Mansour Sobeh, Esraa ElHawary, Herbenya Peixoto, Rola M. Labib, Heba Handoussa, Noha Swilam, Ahmed H. El-Khatib, Farukh Sharapov, Tamer Mohamed, Sonja Krstin, Michael W. Linscheid, Abdel Nasser Singab, Michael Wink, Nahla Ayoub

**Affiliations:** 1 Institute of Pharmacy and Molecular Biotechnology, Heidelberg University, Heidelberg, Germany; 2 Department of Pharmacognosy, Faculty of Pharmacy, Ain Shams University, Cairo, Egypt; 3 Department of Pharmaceutical Biology, Faculty of Pharmacy and Biotechnology, German University in Cairo, Cairo, Egypt; 4 Department of Pharmacognosy, Faculty of Pharmacy, British University in Egypt, Cairo, Egypt; 5 Department of Chemistry, Humboldt Universität Berlin, Berlin, Germany; 6 Pharmaceutical Analytical Chemistry Department, Faculty of Pharmacy, Ain Shams University, Cairo, Egypt; 7 Department of Pharmacology and Toxicology, Faculty of Medicine, Umm Al-Qura University, Makkah, Saudi Arabia

**Keywords:** Polyphenolics, LC/HRESI/MS/MS, *Caenorhabditis elegans*, Antioxidant activity., *Schotia brachypetala*

## Abstract

**Background:**

*Schotia brachypetala* Sond. (Fabaceae) is an endemic tree of Southern Africa whose phytochemistry and pharmacology were slightly studied. The present work aimed at profiling the major phenolics compounds present in the hydro-alcohol extract from *S. brachypetala* leaves (SBE) using LC/HRESI/MS/MS and NMR and prove their antioxidant capabilities using novel methods.

**Methods:**

*In vitro* assays; DPPH, TEAC persulfate decolorizing kinetic and FRAP assays, and *in vivo* assays: *Caenorhabditis elegans* strains maintenance, Intracellular ROS in *C. elegans*, Survival assay, GFP expression and Subcellular DAF-16 localization were employed to evaluate the antioxidant activity.

**Results:**

More than forty polyphenols, including flavonoid glycosides, galloylated flavonoid glycosides, isoflavones, dihydrochalcones, procyanidins, anthocyanins, hydroxy benzoic acid derivatives, hydrolysable tannins, and traces of methylated and acetylated flavonoid derivatives were identified. Three compounds were isolated and identified from the genus *Schotia* for the first time, namely gallic acid, myricetin-3-*O*-*α*-L-^1^*C*_4_-rhamnoside and quercetin-3-*O*-L-^1^*C*_4_-rhamnoside. The total phenolics content of SBE was (376 mg CAE/g), followed by flavonoids (67.87 QE/g). *In vitro* antioxidant activity of SBE was evidenced by DPPH radical scavenging activity (IC_50_ of 9 µg/mL), FRAP ferric reducing activity (5,000 mol Fe^2+^ E/mg) and ABTS peroxide inhibiting activity (1,054 mM Trolox E/mg). The tested extract was able to protect the worms against juglone induced oxidative stress, an increased survival rate (up to 41%) was recorded, when compared with the control group (11%) and attenuate the reactive oxygen species (ROS) accumulation in dose-dependent and reached up to 72% for the highest tested concentration. SBE was also able to attenuate the levels of heat shock protein (HSP) expression in dose-dependent up to 60% in the 150 µg SBE/mL group. In DAF-16 Subcellular localization SBE treated worms showed nuclear localization pattern up to 78%, while it was only 5% in the untreated control group.

**Discussion:**

A pronounced antioxidant activity *in vivo*, which can be attributed to its ability to promote the nuclear translocation of DAF-16/FOXO, the main transcription factor regulating the expression of stress response genes. The remarkable antioxidant activity *in vitro* and *in vivo* correlates to SBE rich phenolic profile.

## Introduction

Plants produce a wide diversity of secondary metabolites, which have evolved as defence compounds against herbivores and microbes. Most secondary metabolites exhibit an interesting pharmacological activity. Therefore, many plants have been used in traditional medicine and phytomedicine for the treatment of health disorders all over the world (Wyk & Wink, 2004). In modern medicine, plants still have a special participation; anticancer compounds such as vinblastine, paclitaxel and camptothecin can be cited as enthusiastic examples of the pharmaceutical potential of the natural products (Efferth & Wink, 2010) Anti-aging, antioxidants and anti-inflammatories are also currently found in natural sources (Angerhofer, Maes & Giacomoni, 2008; Debnath, Kin & Lim, 2013; Kim et al., 2004; Yuan et al., 2006).

Antioxidant compounds are being extensively studied; they are supposed to play a role on aging and aging related diseases due to their ability to attenuate the cellular oxidative damage which are caused essentially by the reactive oxygen species (ROS) (Barja, 2004; Shaw, Werstuck & Chen, 2014).

The production of ROS is an inevitable result of the cell metabolism which can be enhanced by endogenous and exogenous stress. High concentrations of ROS cause oxidative damage on DNA, lipids and proteins; as a consequence, quite a number of health disorders are related to ROS intracellular imbalance, including arteriosclerosis and other cardio-vascular conditions, inflammation, cataract as well as Alzheimer’s disease (Dumont & Beal, 2011; Pendergrass et al., 2006) and even cancer (Valko et al., 2004; Valko et al., 2007).

The cellular defence system against radicals include antioxidant enzymes, like superoxide dismutase, glutathione and catalase and compounds with antioxidant activity like proteins, vitamins, minerals and polyphenols (Sies & Stahl, 1995). ECGC and resveratrol are examples of polyphenols with potent antioxidant activity and demonstrated health benefits (Fujiki et al., 1999; Patel et al., 2010; Rossi et al., 2008; Widlansky et al., 2007; Wolfram, 2007).

*Schotia brachypetala* Sond. (Fabaceae), commonly named weeping boer-bean and huilboer-bean (Afrikaans), is a tree endemic to southern Africa (Brenan, 1867; Watt & Breyer-Brandwijk, 1932). Polyhydroxystilbenes were isolated from the heartwood of the tree (Drewes & Fletcher, 1974) and two antibacterial fatty acids [methyl-5,11,14,17-eicosatetraenoate and 9,12,15-octadecatrienoic (*δ*-linolenic acid)] have been described from the leaves (McGaw, Jäger & Staden, 2002). Flavonolacyl glucosides were recently reported from aerial parts of *S. brachypetala* (Du et al., 2014). A recent report indicates the identification of polyphenols from the stalks of *S. Brachypetala* for the first time namely, daidzein, naringin, procyanidin isomers, procyanidin dimer gallate, quercetin 3-*O*-glucuronide, quercetin hexose gallic acid, quercetin hexose protocatechuic acid, and ellagic acid. The results of this study provide evidence that the phenolic rich extracts of *S. brachypetala*, *Camellia sinensis*, *Markhamia platycalyx*, and piceatannol have high potential to be anti-Alzheimer’s disease drug leads (Hassaan et al., 2014). In addition, catechin and epicatechin have been isolated from the bark of *Schotia latifolia* Jacq (Masika, Sultana & Afolayan, 2004).

Traditional healers applied a decoction of the bark to strengthen the body and to treat dysentery and diarrhoea, nervous and heart conditions, flu symptoms and as an emetic. The roots are also used to treat diarrhoea and heartburn. The seeds can be roasted and eaten (Du et al., 2014). Extracts from various parts of *S. brachypetala* were active against bacteria that cause gastrointestinal infections; this would explain the use of this plant in the traditional treatment of diarrhoea (Paiva et al., 2010). Furthermore, these extracts showed anti-oxidant, anti-bacterial and anti-malarial activities (Du et al., 2014), and were active against Alzheimer’s disease, which was correlated to their anti-oxidant and probably anti-inflammatory properties (Hassaan et al., 2014).

The current work aimed to characterize the phenolic secondary metabolites of *S. brachypetala* leaves using LC/HRESI/MS/MS and NMR. To evaluate its antioxidant activity *in vivo*, the nematode *Caenorhabditis elegans* was used, since it is a well-established model suitable to study stress resistance, aging, and longevity.

## Materials and Methods

### Plant material

During the spring season (April–May 2012) *S. brachypetala* leaves were collected from trees grown in Orman Botanical Garden, Dokki, Giza, (Arab Republic of Egypt). The authenticity of the species was confirmed by Professor Dr. Mohamed El Gebaly (Professor of Taxonomy at the National Research Centre, Egypt). The identity was further confirmed by DNA barcoding which was carried in our laboratory using *rbc*L as a marker gene to confirm the *Schotia* species. A voucher specimen was deposited at the herbarium of department of pharmacognosy, Faculty of Pharmacy, Ain Shams University, Egypt. Leaves sample was kept under voucher number P8563/12 at IPMB drug store. Specific permission was not required for research purpose because the plant was grown as an ornamental tree in the Botanical Garden. The authors confirm that the field studies did not involve endangered or protected species.

### Plant material, extraction and isolation

*S. brachypetala* leaves (1 kg) were exhaustively extracted with distilled water (5 Lx3). At low temperature, the extract was dried under vacuum followed by alcohol extraction. Similarly, the soluble alcohol extract was dried under vacuum. SBE dried powder of the aqueous alcohol (30 g) was fractionated by column chromatography using polyamide S6 column. Gradient elution was carried out to obtain four main fractions. Fraction II showed only one major spot and was compared to reference gallic acid, Fraction III was applied on top of Sephadex LH-20 column and eluted using the solvent butanol saturated with water to yield chramatgraphically pure samples of compounds 1 and 2; Fraction IV was purified using PPC (preparative paper chromatography). Both Fraction III and IV were subjected to further analysis by LC/ESI/MS^n^. Compounds isolated from fractions III were analyzed using ^1^H-NMR spectroscopy.

### Solvents, chemicals and apparatus

HPLC analysis was performed using HPLC grade solvents. All other chemicals used in the current work in the isolation of the compounds and in the biological assays were purchased from Sigma-Aldrich Chemicals with analytical grade. Spectrophotometer (Tecan Group Ltd., Männedorf, Switzerland) and Fluorescence microscope BIOREVO BZ-9000 (Keyence Deutschland GmbH, Neu-Isenburg, Germany) were used in biological assay.

### LC–HRESI-MS–MS

The chromatographic analysis was performed on an HPLC Agilent 1200 series instrument, the column was Gemini 3 µm C18 110A° from Phenomenex with dimensions 100 × 1 mm i.d., protected with RP C18 100 A° guard column with dimensions (5 mm × 300 µm i.d., 5 µm). The mobile phase was consisted of two solvents (A) 2% acetic acid and (B) 90% MeOH, 2% acetic acid at a flow rate of 50 µL/min. The sample was dissolved in 5% MeOH and 2% acetic acid while the sample injection volume was 10 µL. A Fourier transform ion cyclotron resonance mass analyzer was used equipped with an electrospray ionization (ESI) system. X-calibur^®^ software was used to control the system. Detection was performed in the negative ion mode applying a capillary voltage of 36 V and a temperature of 275 °C. The API source voltage was adjusted to 5 kV, and the desolvation temperature to 275 °C. Nitrogen was used as a nebulizing gas with a flow adjusted to 15 L/min. The analytical run time was 89 min and the full mass scan covered the mass range from 150 to 2,000 *m*∕*z* with resolution up to 100,000 (Shaw, Werstuck & Chen, 2014).

### NMR

For ^1^H-NMR experiments, samples were dissolved in deuterated DMSO-d_6_ and measured in 5 mm tubes at 25  °C on a BRUKER 400 MHz NMR spectrometer.

### HPLC standardization of SBE

The hydro-alcohol extract (SBE) was standardized using an Agilent 1200 series HPLC instrument equipped with an Agilent quaternary pump connected to a photodiode array detector (PDA) with variable wavelengths. The separation was performed on a RP-C18 column with the following dimensions: 150 mm, 4.6 mm, 5 µm. The standard used was gallic acid (Sigma-Aldrich Chemicals) prepared in a dilution of 1.296 mg/mL in HPLC grade methanol to give a stock solution from which serial dilutions were prepared (0.001, 0.002, 0.003 and 0.004 mg/mL). All samples were tested using 4% acetic acid/ water (solvent A) and methanol (solvent B) in gradient program. The gradient program was 0–4 min 100% A, 4.01–10 min 50% A in 50% B, 10–20 min 20% A in 80% B, 20–22 min 50% A in 50% B, 22–26 min 100% B, with flow rate 0.6 mL/min. 20  µL was injected onto the chromatograph, the detection was carried out at 280 nm wavelength (Mradu et al., 2012). Different concentrations of the reference standard were plotted against the peak area to establish the calibration curve.

### Antioxidant activity *in vitro*

#### DPPH^•^ assay

The radical scavenging activity of SBE was assessed using the stable free radical DPPH^•^ (2,2-diphenyl-1-picrylhydrazyl). The assay was performed according to the standard technique described by Blois (1958) with some modifications to a 96-well microplate. In brief, 100 µL of DPPH solution (200 µM) were added to 100 µL of the SBE with concentrations ranges between (50–1.25 µg/mL). In the dark at room temperature, the samples were incubated for 30 min. The absorbance was measured at 517 nm. The ability of the samples to scavenge the DPPH radicals was calculated according to the following equation:

\begin{eqnarray*}\text{DPPH scavenging effect}~(\text{%})=[(\text{A0}-\text{A1})/\text{A0}]\times 100 \end{eqnarray*}

where A0 represents the control absorbance, and A1 the absorbance of SBE. All measurements were performed in triplicate. The EC_50_ value (µg SBE/mL) was estimated by sigmoid non-linear regression using adequate software.

#### TEAC persulfate decolorizing kinetic assay

Trolox equivalent antioxidant capacity (TEAC) assay uses green-coloured cation radicals of ABTS [2,2′-azinobis-(3-ethylbenzothiazoline-6-sulfonic acid)]. The assay was carried out to assess the quenching ability of the compounds in relation to the reactivity of Trolox, a water-soluble vitamin E analogue. TEAC assay was performed as described by Re et al. (1999) adapted to a 96-well microplate. Initially, the reaction between 7 mM ABTS^•+^ and 2.45 mM potassium persulfate in water (final concentration) was used to generate ABTS^•+^ radical. The reaction was kept for 12–16 h (stock solution) in the dark and at room temperature. The ABTS^•+^ working solution was prepared in water. The absorbance of the working solution was (*A*_734_ = 0.7 ± 0.02). Trolox stock solution (11.5 mM) was prepared in ethanol and then diluted in water to give the working solution. 50 µL of Trolox or SBE were added in each individual well. Consequently, 250 µL of ABTS^•+^ working solution was added. The samples were kept for 6 min at room temperature, and then the absorbance was measured at 734 nm using a spectrophotometer plate reader. All measures were performed in triplicate and repeated at least three times. The results were expressed in Trolox equivalent/mg of sample.

#### FRAP assay

FRAP assay, Ferric Reducing Antioxidant Power, was performed as previously reported by Benzie & Strain (1996) adapted to a 96-well microplate. The assay depends on the ability of the extract to reduce the ferric complex (2,4,6-tripyridyl-*s*-triazine-Fe^3+^-TPTZ) to its ferrous form (Fe^2+^-TPTZ) at low pH. 300 mM acetate buffer at pH 3.6, 10 mM TPTZ (2,4,6-tripyridyl-*s*-triazine) in 40 mMHCl and 20 mM FeCl_3_.6H_2_O were used to prepare the FRAP working solution by mixing them in the ratio 10:1:1 prior to analysis. The fresh FRAP working solution was warmed to 37  °C for 30 min prior to the assay. FeSO_4_.7H_2_O was used as standard.

A freshly prepared FRAP working solution (175 µL) was added to the samples (25 µL), the reaction was kept for 7 min at 37 °C. All measurements performed in triplicate and repeated three times. As a colorimetric assay, the reduction is indicated by development of an intense blue colour measured at 595 nm using a spectrophotometer microplate reader. FRAP values were showed as molFe(II)/mg of SBE sample.

### Antioxidant activity *in vivo*

#### *Caenorhabditis elegans* strains and maintenance

Nematodes were maintained under standard conditions (on nematode growth medium—NGM—inoculated with living *E. coli* OP50, and incubated at 20 °C). Age synchronized cultures were obtained by sodium hypochlorite treatment of gravid adults; the eggs were allowed to hatch in M9 buffer and larvae obtained were subsequently transferred to S-medium inoculated with living *E. coli* OP50 (D.O_600_ = 1.0) (Stiernagle, 2009). In the current work the following *C. elegans* strains were used: Wild type (N2), TJ375 [*hsp-16.2::GFP(gpls1)*] and TJ356. All of them provided by the *Caenorhabditis* Genetic Center (CGC).

#### Survival assay under juglone-induced oxidative stress

Synchronized worms (L1 larvae stage, N2 strain grown at 20 °C in S-media inoculated with living *E. coli* OP50 − D.O_600_ = 1.0) were treated with 50 µg, 100 µg and 150 µg SBE/mL for 48 h, except the control group. Then, juglone 80 µM was added as a single dose to the medium. 24 h after of the juglone treatment, the survivors were counted (Abbas & Wink, 2014). The result is presented as percentage of live worms, compared by one-way ANOVA followed by Bonferroni (post-hoc) correction.

#### Intracellular ROS in *C. elegans*

Synchronized worms (L1 larvae stage, N2 strain grown at 20 °C in S-media inoculated with living *E. coli* OP50 − D.O_600_ = 1.0) were treated with 50 µg, 100 µg and 150 µg SBE/mL for 48 h, except the control group. After treatment, the worms were carefully washed in M9 buffer and then transferred to 1 mL of CM-H_2_DCF-DA 20 µM and incubated for 30 min at 20 °C. To remove the excess of dye, the worms were washed once more with M9 buffer and finally analysed by fluorescence microscopy (*λ*_*Ex*_ 480/20 nm; *λ*_*Em*_ 510/38 nm). The worms were paralyzed with sodium azide 10 mM and placed on a glass slide. Images were taken from at least 30 worms at constant exposure time. The relative fluorescence of the whole body was determined densitometrically using Image J software. The results are shown as mean pixel intensity (mean ± SEM) and compared by one-way ANOVA followed by Bonferroni (post-hoc) correction.

#### Quantification of hsp-16.2::GFP expression

Synchronized transgenic *C. elegans* TJ375 [expressing *hsp-16.2::GFP(gpls1)*] were grown at 20 °C in S media with living *E. coli* OP50 (D.O_600 nm_ = 1.0). L4 worms were treated for 48 h with 50, 100 and 150 µg SBE/mL, except the control group. Then they were exposed to juglone 20 µM for 24 h and finally analysed by fluorescence microscopy (*λ*_*Ex*_ 480/20 nm; *λ*_*Em*_ 510/38 nm). The mutant strain contains *hsp-12.6* promoter coupled to the gene encoding GFP (green fluorescence protein), whose expression is directly quantified by observing the fluorescence intensity of the GFP reporter in the pharynx of the worm. The worms were paralyzed with sodium azide 10 mM and placed on a glass slide. Images were taken from at least 30 nematodes using 20X objective lens at constant exposure time. The relative fluorescence of the pharynx was determined densitometrically using imageJ software. The results are shown as mean pixel intensity (mean ± SEM) and then compared by one-way ANOVA followed by Bonferroni (post-hoc) correction.

#### Subcellular DAF-16 l ocalization

Synchronized transgenic TJ356 worms (L1 larvae grown in S media at 20 °C with living *E. coli* OP50 − D.O_600 nm_ = 1.0), which have a DAF-16::GFP fusion protein as reporter, were treated for 72 h with 50, 100 and 150 µg SBE/mL, except the control group. In M9 buffer, the worms were paralyzed with sodium azide 10 mM and placed on a glass slide. Images were taken from at least 30 worms using 10X objective lens at constant exposure time. According to DAF-16::GFP fusion protein major location, the worms were sorted in three categories: cytosolic, intermediate and nuclear. The results are shown as percentage (mean ± SEM) and compared by one-way ANOVA followed by Bonferroni (post-hoc) correction.

## Results and Discussion

### Identification of the isolated flavonoid glycosides by NMR

Two flavonoid glycosides (myrecitin-3-*O*-*α*-L-^1^*C*_4_-rhamnoside) and (quercetin-3-*O*-*α*-L-^1^*C*_4_-rhamnoside), were isolated and identified from SBE for the first time.

Compound **1** (23 mg) was isolated as yellow crystalline powder. On PC, it showed a dark purple spot under short UV light. *R*_*f*_ values: 24.5 (BAW) and 13.5 (6% AcOH). It gave a dirty green colour with FeCl_3_ spray reagent which is specific for phenolics. Also, its UV spectrum showed two bands at *λ*_*max*_MeOH (350 nm band I and 206 nm band II), which are indicative the flavone nucleus. It showed a bathochromic shift (19 nm) on addition of sodium methoxide and (66 nm) in band II with sodium acetate to prove that the 3′, 4′, 5′ and 7 OH positions are free. The ^1^H-NMR spectra indicated the absence of the signal for H-3, the presence of aromatic proton signals at *δ* = 6.15 ppm (1H, *s*, H-8) and *δ* = 6.31 ppm (1H, *s*, H-6), presence of *O*-glycosidic anomeric signal at *δ* = 5.2 ppm (1H, *s*, H-1″) and signal for methyl of rhamnose at *δ* = 1.51 ppm (3H, S, CH_3_ rhamnose). UV as well as ^1^H-NMR chemical shifts were found to be similar to those previously reported for myrecitin-3-*O*-*α*-L-^1^*C*_4_-rhamnoside. Consequently, compound **1** was confirmed to be myrecitin-3-*O*-*α*-L-^1^*C*_4_-rhamnoside (Hayder et al., 2008).

**Compound** **2** (39 mg) was obtained as yellow crystalline powder. On PC, it showed a dark purple spot under short UV light. R_*f*_values: 22.5 (BAW) and 7.5 (6% AcOH). It gave a dirty green colour with the FeCl_3_spray reagent. Also, its UV spectrum showed two bands at *λ*_*max*_MeOH (350 nm band I and 206 nm band II) which indicated the presence of a flavone nucleus. It showed a bathochromic shift (30 nm) on addition of sodium methoxide and (20 nm) in band II with sodium acetate indicating that the 3′, 4″ and 7 OH positions are free. From these data we conclude that compound **2** corresponds to quercetin-3-*O*-*α*-L-^1^*C*_4_-rhamnoside.

The ^1^H-NMR spectrum of compound **2** indicated the absence of the signal for H-3, the presence of aromatic proton signals at *δ* = 7.199 (1H, *d*, *J* = 2.5 Hz, H-2′), *δ* = 6.909 (1H, *dd*, *J* = 2.5 Hz, 8 Hz, H-6′), *δ* = 6.882 (1H, *d*, *J* = 8 Hz, H-5′), presence of *O*-glycosidic anomeric signal at *δ* = 5.214 ppm (1H, *S*, H-1″) and a signal for methyl of rhamnose at *δ* = 1.242 ppm (3H, *s*, CH_3_rhamnose). UV as well as ^1^H-NMR chemical shifts were found to be similar to those previously reported for quercetin-3-*O*-*α*-L-^1^*C*_4_-rhamnoside. Consequently, compound **2** was identified as quercetin-3-*O*-*α*-L-^1^*C*_4_-rhamnoside (Ma et al., 2005).

### Identification of constituents by LC/HRESI/MS/MS

HPLC-MS plays an important role in the separation and identification of complex plant mixtures. Among its main advantages is the high sensitivity and specificity which can be used both for volatile and non-volatile compounds (Dumont & Beal, 2011).

**Figure 1:**
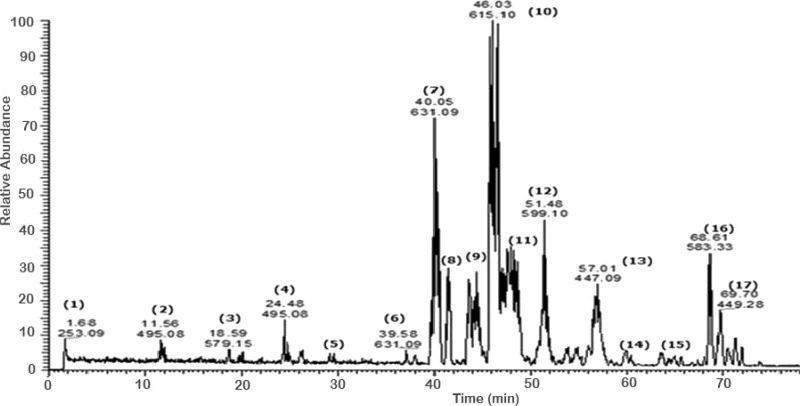
Negative LC/ESI/mass spectrum of phenolics from hydro-alcoholic extract of *Schotia brachypetalea*.

**Figure 2:**
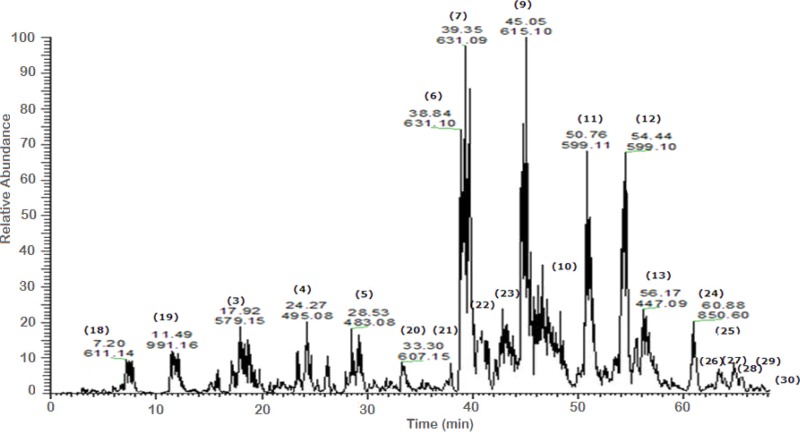
Negative LC/ESI/mass spectrum of phenolics from fraction III of hydro-alcoholic extract of *Schotia brachypetalea*.

**Figure 3:**
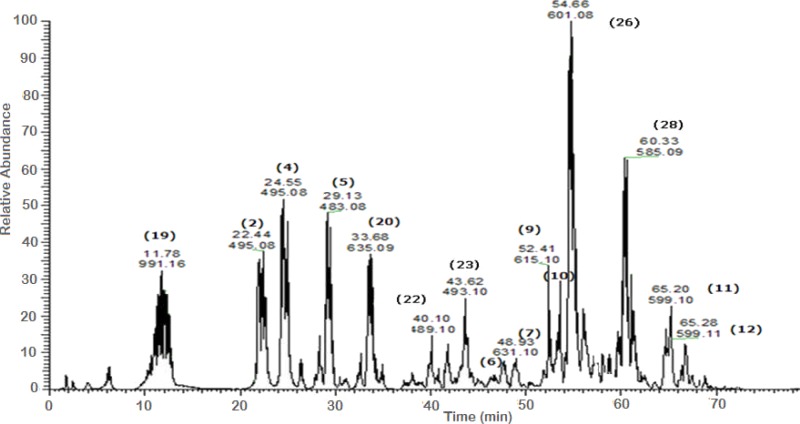
Negative LC/ESI/mass spectrum of phenolics from fraction IV of hydro-alcoholic extract of *Schotia brachypetalea*.

**Figure 4:**
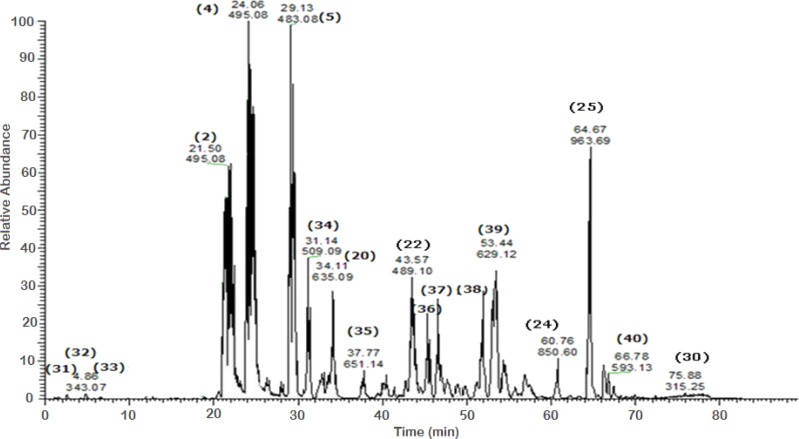
Negative LC/ESI/mass spectrum of phenolics from Sub-fraction I (of fraction 4) of hydro-alcoholic extract of *Schotia brachypetalea*.

**Figure 5:**
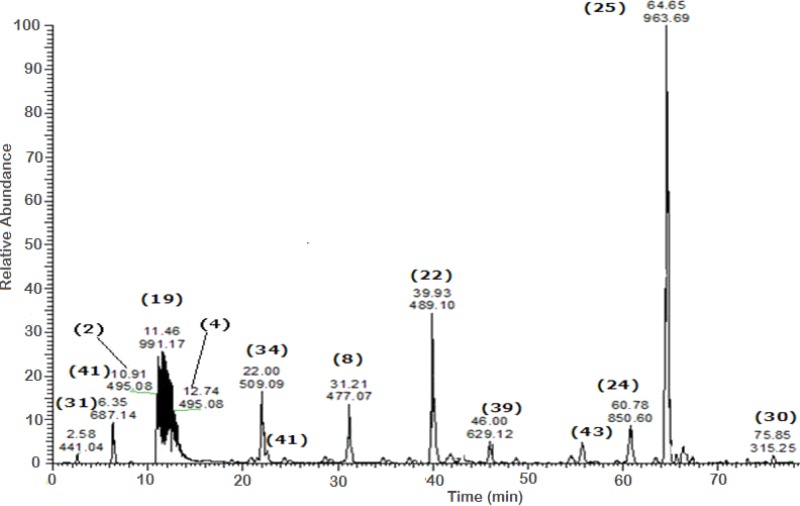
Negative LC/ESI/mass spectrum of phenolics from Sub-fraction II (of fraction 4) of hydro-alcoholic extractof *Schotia brachypetalea*.

A total of 43 secondary metabolites were identified from SBE, its fractions and sub-fractions using LC/ESI/MS/MS (Table 1). LC/HRESI/MS/MS profiles of SBE, its fractions and sub-fractions are shown in Figs. 1–5. Different classes of phenolics were discovered, which will be discussed in the following:

**Table 1:** Compounds identified from the total leaf extract of *Schotia brachypetalea*, its fractions and subfractions.

#	Compound	Class	*t*_*R*_ (min.)	[M-H]^−^ (*m/z*)	MS/MS fragment	Reference	Source (*t*_*R*_ min.)				
							Extract *(peak area %)*	Fr.3	Fr.4	Sub.1	Sub.2
1	Daidzein	Isoflavone	1.68	253	253	Hanganu, Vlase & Olah (2010)	√*(1.32%)*	–	–	–	–
2	Digalloyl quinic acid	Gallotannin	11.56	495	343	Sannomiya, Montoro & Piacent (2005)	√*(1.32%)*	√ (24.27)	√ (10.92)	√ (12.28)	√ (11.46)
3	Narirutin (naringenin-7-*O*-rutinoside)	Flavonoid glycoside	18.5	579	433, 271	Sanchez-Rabaneda et al. (2003)	√*(1.32%)*	√ (18.35)	–	–	
4	Digalloyl quinic acid	Gallotannin	24.48	495	343	Sannomiya, Montoro & Piacent (2005)	√*(1.25%)*	–	√ (12.47)	–	–
5	Digalloyl hexose	Hydrolysable tannin	29.12	483	343	Poay, Kiong & Hock (2011)	√*(1.20%)*	√ (17.12)	√ (29.13)	√ (15.62)	–
6	Myrecitin-3-*O*-(2″-*O*-galloyl)-hexoside	Galloylated flavonoid glycoside	39.92	631	479, 317	Saldanha, Vilegas & Dokkedal (2013)	√*(2.36%)*	√ (38.84)	√ (48.93)	–	–
7	Myrecitin-3-*O*-(2″-*O*-galloyl)-hexoside	Galloylated flavonoid glycoside	40.05	631	479, 317	Saldanha, Vilegas & Dokkedal (2013)	√*(3.98%)*	√ (39.35)	–	–	–
8	Quercetin-3-*O*-glucouronide	Flavonoid	43.62	477	301, 179, 151	Saldanha, Vilegas & Dokkedal (2013)	√*(4.85%)*	√ (42.80)	√ (43.36)	–	√ (31.21)
9	Quercetin-3-*O*-(2″-*O*-galloyl)-hexoside	Galloylated flavonoid glycoside	44.03	615	463, 301	Saldanha, Vilegas & Dokkedal (2013)	√*(12.81%)*	√ (44.72)	√ (47.64)	–	–
10	Quercetin-3-*O*-(2″-*O*-galloyl)-hexoside	Galloylated flavonoid glycoside	46.76	615	463, 301	Saldanha, Vilegas & Dokkedal (2013)	√*(15.75%)*	√ (45.05)	√ (52.41)	–	–
11	Quercetin-hexose-protocatechuic acid	Galloylated flavonoid glycoside	51.48	599	463, 300	Abdel-Hameed, Bazaid & Salman (2013)	√*(7.34%)*	√ (50.76)	√ (65.20)	–	–
12	Quercetin-hexose protocatechuic acid	Galloylated flavonoid glycoside	54.71	599	463, 300	Abdel-Hameed, Bazaid & Salman (2013)	√*(5.62%)*	√ (51.13)	√ (65.28)	–	–
13	Quercetin-3-*O*-rhamnoside	Flavonoid glycoside	57.01	447	301	Saldanha, Vilegas & Dokkedal (2013)	√*(5.72%)*	√ (56.17)	–	–	√ (58.78)
14	Myricetin-3-*O* − *α*-arabinopentoside	Flavonoid glycoside	59.91	449	271, 179	Saldanha, Vilegas & Dokkedal (2013)	√*(2.56%)*	–	–	–	–
15	Kaempferol-3-*O*-rhamnoside	Flavonoid glycoside	63.56	431	285	Diantini, Subarnas & Lestari (2012)	√*(2.75%)*	–	–	–	–
16	Kaempferol derivative	Flavonoid glycoside	68.61	583	285	Saldanha, Vilegas & Dokkedal (2013)	√*(1.29%)*	–	–	–	–
17	Myricetin-3-*O*-*α*-arabinopentoside	Flavonoid glycoside	69.70	449	271, 179	Saldanha, Vilegas & Dokkedal (2013)	√*(4.94%)*	–	–	–	–
18	Unidentified	——	7.1	611	——	——	–	√	–	–	–
19	Pentagalloyl-hexoside	Hydrolysable tannin	11.2	991	495, 343	Poay, Kiong & Hock (2011)	–	√	–	–	–
20	Trigalloyl hexose isomer	Hydrolysable tannin	33.68	635	463, 343, 211, 161	Poay, Kiong & Hock (2011)	–	–	√	√	–
21	1-*O*-galloyl-6-*O*-cinnamoyl-*p*-coumaryl-hexoside	Hydrolysable tannin	33.3	607	461	Tentative	–	√	–	–	–
22	Luteolin-7-*O*-6″-acetylhexoside	Flavonoid	40.10	489	467, 285	Saldanha, Vilegas & Dokkedal (2013)	–	√	–	–	–
23	Caffeoyl-*O*-hexo-galloyl	Hydrolysable tannin	43.62	493	331, 313	Poay, Kiong & Hock (2011)	–	√	–	–	–
24	Procyanidin trimer	Procyanidin	60.88	850	697, 425, 407	Poay, Kiong & Hock (2011)	–	√	–	√ (60.76)	–
25	Methoxylated castalagin/vescalagin	Methyl flavonoid glycoside	64.75	963	933	Rauha, Wolfender & Salminen (2001)	–	√	–	√ (64.67)	√ (64.65)
26	Myrecitin-3-*O*-(2″-*O*-galloyl)-pentoside	Galloylated flavonoid	65.07	601	449	Saldanha, Vilegas & Dokkedal (2013)	–	√	–	–	–
27	Myrecitin-3-*O*-(2″-*O*-galloyl)-pentoside	Galloylated flavonoid	66.02	601	449	Saldanha, Vilegas & Dokkedal (2013)	–	√	–	–	–
28	Quercetin-3-*O*-(2″-*O*-galloyl)-pentoside	Galloylated flavonoid	67.38	585	433, 301	Saldanha, Vilegas & Dokkedal (2013)	–	√	–	–	–
29	Luteolin aglycone	Flavonoid	67.45	285	285	Saldanha, Vilegas & Dokkedal (2013)	–	√	–	–	–
30	Isorhamnetin	Flavonol	67.68	315	301, 151	Sanchez-Rabaneda et al. (2003)	–	√	–	√ (75.88)	–
31	(epi) Catechin gallate	Flavanol	2.58	441	289, 169, 135	Bastos et al. (2007)	–	–	–	√	√ (2.58)
32	Galloyl quinic acid/epiquinic	Hydroxybenzoic acid derivative	4.86	343	191, 85	Clifford, Stoupi & Kuhnert (2007)	–	–	–	√	–
33	Galloyl quinic acid /epiquinic	Hydroxybenzoic acid derivative	6.49	343	191, 85	Clifford, Stoupi & Kuhnert (2007)	–	–	–	√	–
34	Dihydromyricetin methylated dihexoside derivative	Flavonoid dervitative	31.14	509	347	Tentative	–	–	–	√	–
35	Luteolin-7-*O*-hexosyl-8-*C*-(6″-acetyl)-hexoside	Acetyl flavonoid glycoside	37.77	651	489, 327, 179, 151	Simirgiotis et al. (2013)	–	–	–	√	–
36	Isorhamnetin acetyl glucoside	Acetylated flavonoid glycoside	45.36	519	357, 315	Simirgiotis et al. (2013)	–	–	–	√	√ (41.71)
37	Quercetin-3-*O*-hexoside	Flavonoid glycoside	48.87	463	301	Sannomiya, Montoro & Piacent (2005)	–	–	–	√	–
38	Quercetin-3-*O*-hexohexoside	Flavonoid glycoside	51.93	463	301	Sannomiya, Montoro & Piacent (2005)	–	–	–	√	–
39	Unidentified	————	53.44	629	———	———	–	–	–	√	–
40	Kaempferol-3-*O*-rutinoside	Flavonoid glycoside	66.78	593	285	Sannomiya, Montoro & Piacent (2005)	–	–	–	√	
41	Luteolin-5-*O*-hexosyl-8-*C*-(6″-acetyl)-hexoside derivative	Acetyl flavonoid glycoside	6.35	687	651, 489, 327	Simirgiotis et al. (2013)	–	–	–	–	√
42	Phloretin xyloglucoside	Dihydrochalcone	21.48	567	435, 273	Balazs et al. (2012)	–	–	–	–	√
43	Procyanidin Dimer-hexoside	Flavonoid glycoside	55.78	737	611,449	Balazs et al. (2012)	–	–	–	–	√
44	Tricin-7-*O*-neohesperidoside	*O*-methylated flavone	59.33	638	492,330	Balazs et al. (2012)	–	–	–	–	√
45	Hesperitin	aglycone	63.44	301	157	Balazs et al. (2012)	–	–	–	–	√

#### Flavonoid glycosides

The negative ion mode profile of LC-ESI-MS/MS showed a major peak (peak area 4.85%) with a [M-H]^−^ at *m*∕*z* 477 representing quercetin-3-*O*-glucouronide **(8)** and a fragment at *m*∕*z* 301 for the deprotonated quercetin aglycone. The difference of 176 mass units indicates a glucuronic acid moiety; the fragment at *m*∕*z* 151 of ring A in quercetin aglycone moiety, confirming the quercetin aglycone identity (Saldanha, Vilegas & Dokkedal, 2013). Another peak for the deprotonated ion *m*∕*z* 447 was identified as quercetin-3-*O*-rhamnoside **(13)** according to literature data (Saldanha, Vilegas & Dokkedal, 2013), accompanied with a fragmentation at *m*∕*z* 301 due to cleavage of the *O*-glycosidic bond releasing free aglycone and loss of a sugar moiety.

Another molecular ion peak (*m*∕*z* 431) was identified as kaempferol-3-*O*-rhamnoside **(15)** (Diantini, Subarnas & Lestari, 2012) with a major fragment at *m*∕*z* 285 corresponding to the kaempferol aglycone (Diantini, Subarnas & Lestari, 2012).

Quercetin-3-*O*-hexoside isomers **(37) (38)** were identified by a molecular peak of *m*∕*z* 463 accompanied by fragment ions at *m*∕*z* 301 indicative for a quercetin aglycone. Flavonol aglycones like quercetin produce a characteristic ion the deprotonated fragment [M–H]^−^, moreover, they produce ions corresponding to retro-Diels-Alder (RDA) fragmentation in the ring C, involving 1,3-scission (Sannomiya, Montoro & Piacent, 2005). Kaempferol-3-*O*-rutinoside **(40)** as an example for flavonol-*O*-dihexosides was identified with *m*∕*z* 593 (Valko et al., 2007), which was further confirmed in comparison with an authentic reference substance.

The pka values for each of the compounds confirmed the sequence of elution all over the peaks. Based on MS–MS fragmentation a [M–H]^−^ signal at *m*∕*z* 519 was assigned to isorhamnetin acetyl-glucoside (an acylated flavonol glycoside) **(36)** which is characterized by the loss of a glucose and a complete acetyl glucose unit, producing fragments with strong intensity at *m*∕*z* 357 [M-162-H] and at *m*∕*z* 315 [M-162–42- H], respectively.

#### Galloylated flavonoid glycosides

A number of galloylated derivatives were identified as major peaks with [M-H]^−^ at *m*∕*z* 631. According to literature data (Saldanha, Vilegas & Dokkedal, 2013), they represent myrecitin-3-*O*-(2″-*O*-galloyl)-hexoside and its isomer **(6) (7)**. Informative ions are: deprotonated molecular mass [M-H]^−^ (*m*∕*z* 631), fragment ion peak for deprotonated myrecitin hexoside (*m*∕*z* 479), and a deprotonated myrecitin at *m*∕*z* 317. Two peaks with the same pattern were detected suggesting the presence of sugar isomers.

Major peaks of quercetin-3-*O*-(2″-*O*-galloyl)-hexoside and its isomer **(9) (10)**, showed deprotonated molecule peak [M-H]^−^ at *m*∕*z* 615, a fragment ion peak for the deprotonated quercetin hexoside (*m*∕*z* 463), and for the deprotonated quercetin aglycone at *m*∕*z* 301 (Saldanha, Vilegas & Dokkedal, 2013).

Additionally, the molecular ion peak at *m*∕*z* 599, which is indicative for the deprotonated quercetin hexose protocatechuic acid and its sugar isomer **(11) (12)**; fragment ions at *m*∕*z* 463 and *m*∕*z* 300 may be due to the loss of the hexose and the protocatechuic acid moiety, respectively (Abdel-Hameed, Bazaid & Salman, 2013). Furthermore, the molecular ion peak [M-H]^−^ at *m*∕*z* 601 and its deprotonated fragment at *m*∕*z* 449 were identified as myrecitin-3-*O*-(2″-galloyl)-pentoside (Saldanha, Vilegas & Dokkedal, 2013), the difference of *m*∕*z* 152 is due to a loss of galloyl moiety from the molecule. The presence of two molecular ion peaks with the same fragmentation pattern but different retention times indicates the presence of isomers. Similarly, the peak at *m*∕*z* 585, with the difference in aglycone moiety (quercetin instead of myrecitin), represents the deprotonated molecular ion of quercetin-3-*O*-(2″-galloyl)-pentoside **(28)** (Saldanha, Vilegas & Dokkedal, 2013) and deprotonated fragments at (*m*∕*z* 433) and (*m*∕*z* 301) suggest the sequential loss of a galloyl and pentose moities, respectively.

#### Hydroxybenzoic acid derivatives

This class was represented by a deprotonated molecular ion peak at *m*∕*z* 343 indicative for galloyl-quinic / epiquinic acid **(32) (33)** and the deprotonated fragments at *m*∕*z* 191, and *m*∕*z* 85; fragment *m*∕*z* 191 being consistent with quinic acid (Clifford, Stoupi & Kuhnert, 2007). The presence of two peaks with *m*∕*z* 343 but different retention times can be explained by the presence of quinic acid and its isomer epiquinic acid **(27) (28)** (Eliwl & Ramirez, 1997).

#### Isoflavones

A minor peak of daidzein aglycone **(1)** was recognized as a deprotonated peak at *m*∕*z* 253.

#### Dihydrochalcones

A hexoside derivative of phloretin, a characteristic and quite common aglycone previously reported in apple, was identified in SBE as phloretin-3-*O*-xyloglucoside **(42)** with *m*∕*z* 567 and a major ion peak at *m*∕*z* 273 corresponding to the aglycone of phloretin (Balazs et al., 2012).

#### Procyanidins

A procyanidin dimer-hexoside **(43)** was identified and recognized at *m*∕*z* 737 with fragmentation pattern as follows: A product ion of *m*∕*z* 611 containing the galactoside was formed by the loss of gallic acid (126 Da). However, the second product ion with *m*∕*z* 449 was detected in the spectrum indicates the loss of both the gallic acid and the sugar moiety (Sies & Stahl, 1995). A procyanidin trimer **(24)** was identified according to its deprotonated base peak at *m*∕*z* 850 and its deprotonated fragments at *m*∕*z* 697, 425 and 407, which are produced by a cleavage of the inter-flavan bond through a quinine-methide (QM) cleavage (Passos, Cardoso & Domingues, 2007) to give (*m*∕*z* 425) then a loss of water molecule to yield *m*∕*z* 407 in agreement with a procyanidin trimer MS fragmentation pathway (Passos, Cardoso & Domingues, 2007).

#### Hydrolysable tannins

For tri-galloyl hexose isomer **(20)** a [M-H]^−^ was identified with *m*∕*z* 635. The contribution of the major peak (*m*∕*z* 483) is due to the presence of a digalloyl-hexose moiety. Besides, two intermediate ions were detected at *m*∕*z* 271 and *m*∕*z* 211. They are indicative for mono- and di-galloyl-hexose; the elimination of a hexose moiety from mono galloyl-hexose was detected which subsequently lead to the formation of the deprotonated gallic acid at *m*∕*z* 169 (Poay, Kiong & Hock, 2011).

Represented by a deprotonated parent ion peak at *m*∕*z* 495 for di-galloyl quinic acid **(2) (4)**, different positional isomers arise from the difference in hydroxyl attachment site giving rise to peaks of same *m*∕*z* value. The identification was done according to the identity of the obtained peaks as follows: a [M–H]^−^ at *m*∕*z* 343 indicates the loss of a galloyl moiety from the parent peak and fragmentation showed fragments at *m*∕*z* 191 and *m*∕*z* 169, corresponding to quinic acid and gallic acid moieties, respectively (Sannomiya, Montoro & Piacent, 2005). Compound **(5)** with *m*∕*z* 483, identified as digalloyl hexose, showed an ion peak typical for the dimer analogue of *m*∕*z* 169 produced by gallic acid.

#### Methyl and acetyl flavonoid glycosides

A peak at *m*∕*z* 963 is typical for deprotonated methoxylated castalagin/vescalagin **(25)** showing a major peak at *m*∕*z* 933, corresponding to the polyphenol castalagin or its isomer vescalagin (Rauha, Wolfender & Salminen, 2001).

Two acetyl flavonoid glycosides were detected luteolin-7-*O*-hexosyl-8-*C*-(6″-acetyl)—hexoside (**35)** with *m*∕*z* 651. The detected fragments at *m*∕*z* 179, 151 provide the evidence that luteolin was the aglycone of compound **(35)** (Simirgiotis et al., 2013). Compound (**41)** with a [M-H]^−^ ion at *m*∕*z* 687 showed fragments at *m*∕*z* 651, 489, 327. These ions match with the MS data previously reported for compound (**41**) [luteolin-5-*O*-hexosyl-8-*C*-(6″-acetyl)-hexoside derivative], full MS at (*m*∕*z* 651) after the loss of 38 amu and thus was tentatively assigned to its analogue luteolin-7-*O*-hexosyl-8-*C*-(6″-acetyl)-hexoside **(35)** (Masika, Sultana & Afolayan, 2004).

#### Methyl flavone, flavanol and flavonol

A methyl-flavone was identified as tricin-7-*O*-neohesperidoside **(44)** from its exact mass (*m*∕*z* 638) [M-H]^−^; by taking into consideration the additional mass of 30 for the extra methoxy group on the [M-H]^−^ ion. The major fragments of **(38)** were at *m*∕*z* 492 and 330 corresponding, respectively, to ions [M-H-146]^−^ and [M-H-146-162]. The losses of 146 and 162 Da are characteristic for rhamnose and glucose moieties, respectively, and the ion at *m*∕*z* 329 is characteristic of the aglycone tricin (Paiva et al., 2010).

A flavanol was represented by a deprotonated parent peak for (*epi*) catechin gallate at *m*∕*z* 441**(31)** and its deprotonated fragments at *m*∕*z* 289, 169 and 135 (Bastos et al., 2007). The fragment at *m*∕*z* 289 for the deprotonated (*epi*) catechin (Ivanova et al., 2011), *m*∕*z* 169 for the galloyl moiety, and *m*∕*z* 135 for ring (A) of flavones nucleus. As an example of the flavonol isorhamnetin **(30)**, a deprotonated molecular ion peak was detected at *m*∕*z* 315 with deprotonated fragments at (*m*∕*z* 301, *m*∕*z* 151) (Sanchez-Rabaneda et al., 2003).

### Standardization of SBE using HPLC

The SBE showed an intense peak at *R*_*t*_ 3.983 min corresponding to gallic acid (identified by peak matching with a gallic acid standard). Through the standardization experiment, it was shown that each mg SBE constitutes 0.0022 mg gallic acid. The calibration curve showed good linearity for gallic acid (reference compound) in the range of 0.3 up to 1 mg/mL with correlation coefficient (R2) 0.999.

### Antioxidant activities *in vitro* and *in vivo*:

#### Antioxidant activity *in vitro*

Total phenolic content of SBE was 376 mg of caffeic acid equivalents (CAE)/g SBE while the total flavonoid content was 67.87 mg (quercetin equivalents)/g SBE. The antioxidant activity of SBE was evaluated *in vitro* using three different assays, DPPH, ABTS and FRAP. These methods are widely employed for the antioxidant activity evaluation of pure compounds, plant extracts, as well as food items because long-lived radicals such as DPPH^•^ and ABTS^•+^ as well as FeSO_4_ are sensitive and reliable (Prior, Wu & Schaich, 2005). All methods revealed a strong antioxidant capacity of SBE (Table 2).

**Table 2:** *In vitro* antioxidant activity of SBE.

	DPPH^*^	FRAP^**^	ABTS^***^
**SBE**	9	5,000	1,054
**EGCG**	3	25,000	5,293

**Notes.**

* IC50 = µg/mL.

** Fe^2+^ equivalents/mg of sample.

*** Trolox equivalents/mg of sample.

### Antioxidant activity *in vivo* in *C. elegans*

#### Survival assay

**Figure 6:**
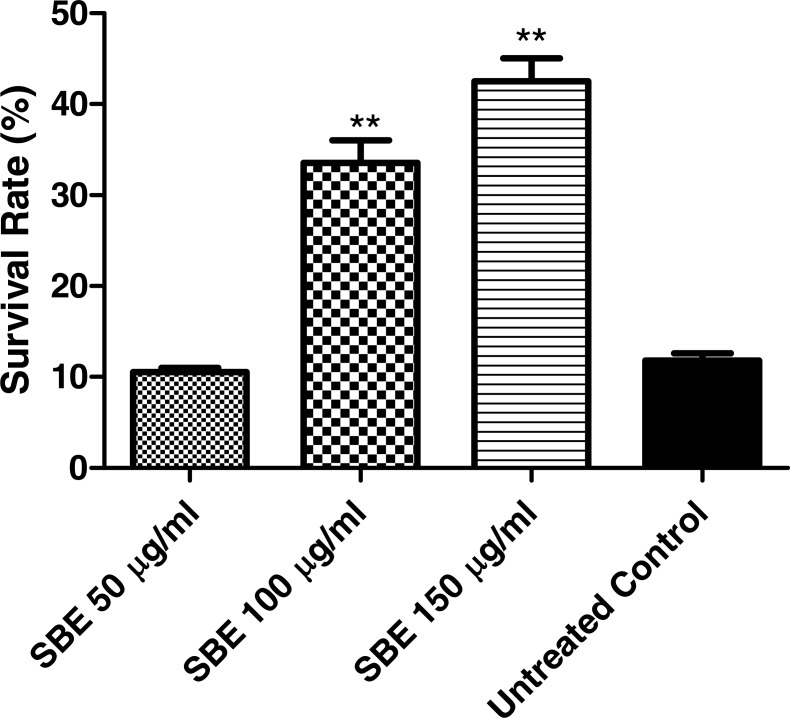
Stress resistance of *C. elegans* under juglone treatment. Survival rates were significantly increased after pre-treatment of the nematodes with SBE. Data are presented as percentage of survivals (mean ± SEM, *n* = 3). ** *p* <  0.01.

Juglone (5-hydroxy-1,4-naphthoquinone) is a natural quinine from *Juglans regia* with toxic pro-oxidant activity (Saling et al., 2011). Exposure of *C. elegans* to a high concentration of juglone kills the worms; however, antioxidant compounds can prevent such an effect. According to our results (Fig. 6), worms pre-treated with SBE showed an increased survival rate (up to 41%), when compared with the control group (11%), which was treated with juglone alone. The increased survival rate indicates that SBE works efficiently as an antioxidant *in vivo*. Similar results have been obtained with other antioxidant polyphenols, such as EGCG from green tea, anthocyanins from purple wheat and aspalathin from Rooibos tea (Abbas & Wink, 2014; Chen et al., 2013).

#### Influence of SBE on intracellular ROS in *C. elegans*

**Figure 7:**
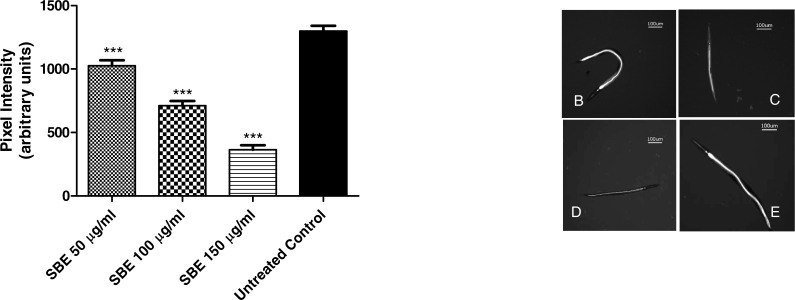
Effect of SBE on intracellular ROS accumulation in *C. elegans*. Data are presented as pixel intensity ± SEM (*n* = 40, replicated 3 times). *** *p* < 0.001 related to the control by a one-way ANOVA followed by Bonferroni (post-hoc) correction.

To assess the intracellular concentration of ROS (reactive oxygen species) and to evaluate a potential antioxidant activity *in vivo*, the membrane permeable reagent 2′,7′- dichlorofluorescin diacetate (CMH_2_DCF-DA) was used. The reagent becomes deacetylated to a non-fluorescent compound by intracellular esterases. The deacetylated form is oxidized in the presence of ROS, especially H_2_O_2_, forming highly fluorescent compound 2′, 7′- dichlorofluorescein (DCF) which can be analysed by fluorescence microscopy. In our experiments, worms were treated for 48 h with three different concentrations of SBE (50, 100 and 150 µg/mL) and then analysed by fluorescence microscopy. The images reveal that the SBE treated worms exhibited significantly lower fluorescence intensity in comparison to the untreated control group (Fig. 7). The decrease in the fluorescence, measured through pixel intensity, was dose-dependent and reaches up to 72% for the highest tested concentration, indicating that SBE is capable to effectively scavenge the ROS *in vivo*.

#### Quantification of *hsp-16.2*:: GFP expression via fluorescence microscopy

Heat shock proteins (HSPs) are virtually found in all living organisms. Increase in HSP levels correlates with exposure to environmental stress conditions that can induce protein damage such as high temperature and presence of oxidants. HSPs play an important role for aging and longevity (Swindell, 2009).

**Figure 8:**
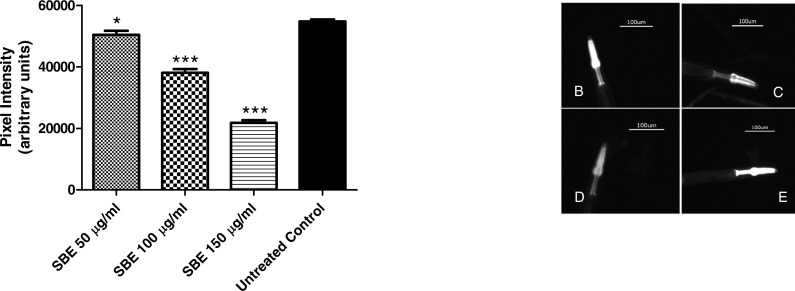
Influence of SBE on hsp16.2::GFP expression in the transgenic *C. elegans* strain (TJ375 hsp-16.2::GFP(gplsI) under juglone-induced oxidative stress. Data are presented as pixel intensity(mean ± SEM, *n* = 40, replicated 3 times). * *p* < 0.05 and *** *p* < 0.001.

To assess the ability of SBE to suppress *hsp*-16.2::GFP expression, worms from the mutant strain TJ375 were used. *hsp*-16.2::GFP expression was induced by juglone treatment. Results revealed that those worms pre-treated with SBE had a significantly lower expression of *hsp-*16.2::GFP, monitored by fluorescence microscopy. The reduction of *hsp*-16.2::GFP expression was dose-dependent and up to 60% in the 150 µg SBE/mL group, in comparison with the control group (Fig. 8). These findings correlate with the demonstrated ability of SBE in increasing the mean survival rate in response to acute oxidative stress (caused by juglone; Fig. 6) and suppress ROS formation *in vivo* (Fig. 8). Similar results have been reported for other phenolic antioxidants, such as EGCG (Abbas & Wink, 2014).

#### Subcellular localization of DAF-16

DAF-16 is a fork head transcription factor (FOXO) family member, present in its phosphorylated form; it remains arrested in the cytosol (inactive form). The dephosphorylated active form migrates into the nucleus and triggers the activity of several target genes related to oxidative stress response and lifespan regulation in both, *C. elegans* and mammals (Mukhopadhyay, Oh & Tissenbaum, 2006).

**Figure 9:**
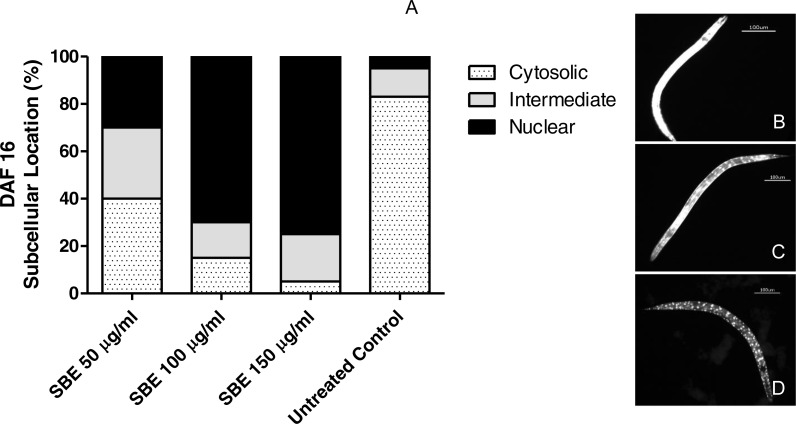
Effect of the leaf extract from *S. brachypetala* (SBE) on DAF-16 subcellular pattern of location in the transgenic *C. elegans* strain (TJ356). Data show the percentage of worms exhibiting cytosolic, intermediate or nuclear pattern of location (A). *** *p* < 0.001 related to the control, analysed by one-way ANOVA followed by Bonferroni (post-hoc) correction. Micrographs illustrate representative location of DAF-16 in the cytosol (B), in cytosol and nucleus(C) and only in the nucleus (D).

In another set of experiments, we investigated whether the antioxidant effects observed, were related to DAF-16/FOXO translocation into the nucleus. Worms (transgenic strain TJ356) were treated with SBE and submitted later to fluorescence microscopy. As illustrated in Fig. 9, a high percentage of the treated worms showed nuclear localization pattern of DAF-16/FOXO (up to 78%), while in the untreated control group, only 5% of the worms exhibited a nuclear localisation pattern. This finding strongly suggests that the ability of SBE to enhance oxidative stress resistance in *C. elegans* is DAF-16/FOXO dependent, similar to the situation with other phenolic antioxidants (Abbas & Wink, 2014; Chen et al., 2013).

## Conclusions

The current study resulted in the identification of different phenolic metabolite classes including flavonoid glycosides, procyanidins, anthocyanins, dihydrochalcones, and hydroxy benzoic acid derivatives. Myricetin-3-*O*-*α*-L-^1^*C*_4_-rhamnoside, quercetin-3-*O*-L-^1^*C*_4_-rhamnoside, and gallic acid were isolated and identified for the first time from the leaves of *S. brachypetala*.

SBE is rich in phenolics, especially flavonoid glycosides such as quercetin which are known as powerful antioxidants *in vitro* (Boukatib, Atmani & Rolando, 2002). Potential health effects of polyphenols have been discussed: several studies reported the ability of quercetin to ameliorate pathological conditions linked to ROS such as oxidation of LDL-cholesterol, to counteract cardiovascular risks (Chopra et al., 2000), to protect primary neurons against A*β* deposits (Ansari et al., 2009). Furthermore, antioxidants are beneficial for chronic inflammation (Comalada et al., 2005; Shoskes et al., 1999) and can avoid Ca^2+^-dependent cell death (Sakanashi et al., 2008)

Our study showed that SBE exhibits a strong antioxidant activity *in vitro* as well as *in vivo*. It is able to decrease ROS production and attenuates *hsp16.2* expression under oxidative stress conditions in *C. elegans*. We assume that a modulation of the DAF-16/FOXO transcription factor by the phenolics is responsible for the observed antioxidant effects. The leaf extract can increase the nuclear location of DAF-16, thereby activating many important biological processes including target genes related to stress resistance and longevity.

Further *in vivo* experiments are needed to develop the polyphenols of *S. brachypetala* into a useful nutraceutical compounds.

##  Supplemental Information

10.7717/peerj.2404/supp-1 Data S1 Tables of raw data for statistics Tables of raw data in collected table for reviewer

10.7717/peerj.2404/supp-2 Supplemental Information 1 HNMR of compound 1

10.7717/peerj.2404/supp-3 Supplemental Information 2 HMNR od compound 2

## Additional Information and Declarations

### Data Availability

The following information was supplied regarding data availability: The raw data has been supplied as a Supplemental Information.
