# Supplementary figures and images for: Identification of phenolic secondary metabolites from Schotia brachypetala Sond. (Fabaceae) and demonstration of their antioxidant activities in Caenorhabditis elegans
Source: PeerJ. 2016 Nov 15;4:e2404. doi: 10.7717/peerj.2404 (PMC5119267; doi:10.7717/peerj.2404)

myrecitin-3-*O*- $\alpha$ -L-<sup>1</sup>C<sub>4</sub>-rhamnoside

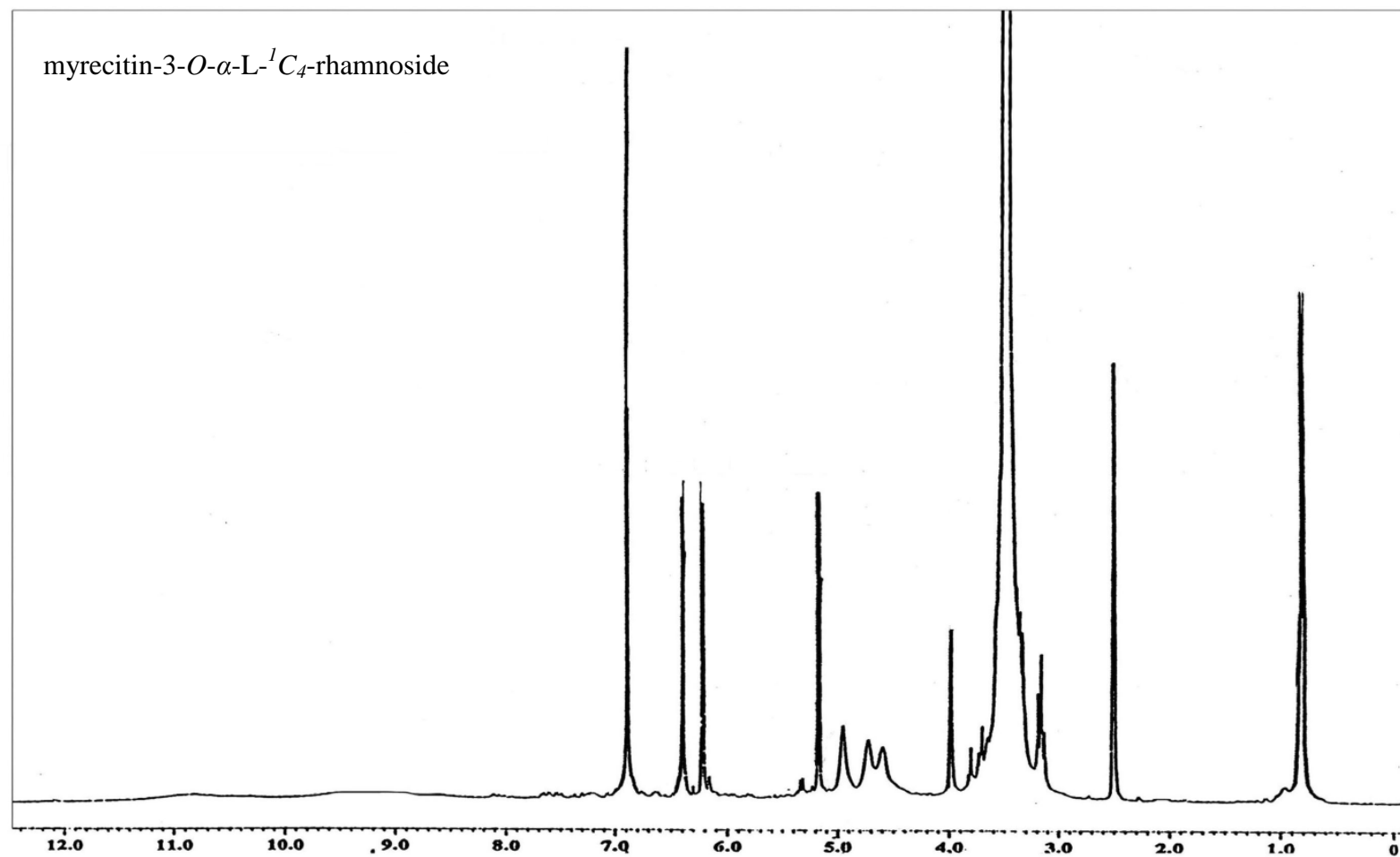

Supplement: Supplemental Information 1 [file peerj-04-2404-s002.pdf]

quercetin-3-*O*- $\alpha$ -L-<sup>1</sup>C<sub>4</sub>-rhamnoside

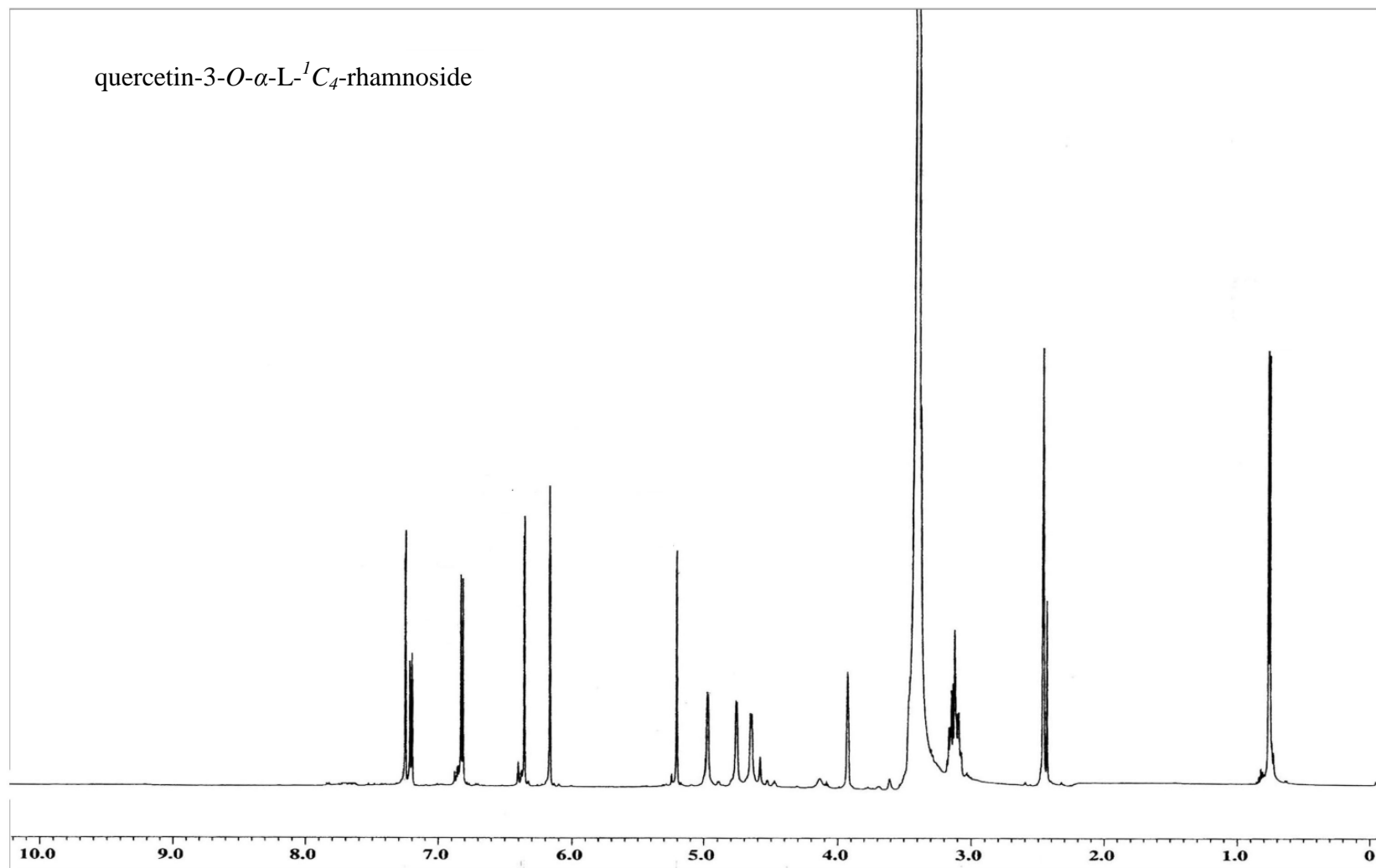

Supplement: Supplemental Information 2 [file peerj-04-2404-s003.pdf]
